# New Biomarkers Based on Dendritic Cells for Breast Cancer Treatment and Prognosis Diagnosis

**DOI:** 10.3390/ijms24044058

**Published:** 2023-02-17

**Authors:** Fanjia Zhao, Fang Yan, Haihong Liu

**Affiliations:** 1 Department of Mathematics, Yunnan Normal University, Kunming 650500, China; 2 Key Laboratory Complex System Modeling and Application for Universities in Yunnan, Kunming 650500, China

**Keywords:** breast cancer, immunotherapy, dendritic cells, prognosis, bioinformatics analysis

## Abstract

Dendritic cells(DCs) play a protective role in the antitumor immunity of most cancers, which can be divided into conventional dendritic cells (cDCs) and plasmacytoid dendritic cells (pDCs). Most current studies are only based on either cDCs or pDCs for the study of the relationship between DCs and breast cancer prognosis, without combining the two together. We aimed to select new biomarkers from pDCs and cDCs. In this paper, the xCell algorithm was first used to calculate the cellular abundance of 64 types of immune cells and stromal cells in tumor samples from the TCGA database, and the high-abundance pDC group and cDC group were divided according to the results of a survival analysis. Then, we looked for the co-expressed gene module of highly infiltrating pDC and cDC patients with a weighted correlation network analysis (WGCNA) and screened out the hub genes, including *RBBP5*, *HNRNPU*, *PEX19*, *TPR*, and *BCL9*. Finally, we analyzed the biological functions of the hub genes, and the results showed that *RBBP5*, *TPR*, and *BCL9* were significantly related to the immune cells and prognosis of patients, and *RBBP5* and *BCL9* were involved in responding to TCF-related instructions of the Wnt pathway. In addition, we also evaluated the response of pDCs and cDCs with different abundances to chemotherapy, and the results showed that the higher the abundance of pDCs and cDCs, the higher their sensitivity to drugs. This paper revealed new biomarkers related to DCs—among them, *BCL9*, *TPR*, and *RBBP5* were proven to be closely related to dendritic cells in cancer. For the first time, this paper puts forward that *HNRNPU* and *PEX19* are related to the prognosis of dendritic cells in cancer, which also provides new possibilities for finding new targets for breast cancer immunotherapy.

## 1. Introduction

Breast cancer is a pathological state in which mammary epithelial cells proliferate uncontrollably under the action of various carcinogens. The tumor microenvironment (TME) has been the focus of tumor-prognosis-related research in recent years; immune cells and stromal cells are two important components of the TME. Studies have shown that the infiltration of immune cells into the TME leads to host–tumor interactions. Immune cells play an integral role in cancer progression and response to therapy, and they are an important prognostic factor for cancer [1,2,3]. Tumor immunotherapy was rated as the most important scientific breakthrough of the year by *Science* magazine in 2013, as it showed strong antitumor activity in breast cancer, liver cancer, ovarian cancer, and other tumor treatments [4,5]. Several tumor immunotherapy drugs have been approved for clinical use by the US Food and Drug Administration (FDA) [6].

Dendritic cells (DCs) are the most functional and professional antigen-presenting cells in the body, and they specialize in triggering adaptive immune responses by activating T cells. Studies have shown that DCs play a protective role in antitumor immunity in most cancers and are currently emerging as promising targets for antitumor therapy [7,8,9,10]. Various subpopulations of DCs are defined according to their ontogeny, phenotype, and anatomical location, and they can be divided into conventional dendritic cells (cDCs) and plasmacytoid dendritic cells (pDCs) [11]. cDCs are specialized antigen-presenting cells that promote naive T cell differentiation and induce cancer immunity. pDCs can produce extremely large amounts of type I interferon (IFN) and play a role in antitumor immunity [12]. There have been many studies about the antitumor effects of cDCs, but only recently has there begun to be a focus on the relevant mechanisms of pDCs in antitumor immunity [13]. Paula Michea et al. pointed out that pDCs are the most distinct subtype of antigen-presenting cells [14]. Tian et al. also found that elevated pDC levels in breast cancer are associated with long-term survival outcomes of patients [15]. In particular, in triple-negative breast cancer, Masanori Oshi found that the ratio of cDCs and pDCs in TNBC was higher than that of other subtypes through a calculation with the xCell algorithm, and high numbers of pDCs were significantly associated with a better survival rate in TNBC [16]. These studies showed that DCs—especially pDCs—play a pivotal role in tumor immunotherapy for breast cancer.

Most of the current studies are only based on either cDCs or pDCs when studying the relationship between DCs and breast cancer prognosis, without combining the two together. This study aimed to select the important genes of DCs from pDCs and cDCs and to evaluate the role of DCs in breast cancer treatment. First, we calculated the cellular abundance of 64 immune cells and stromal cells in tumor samples by using the xCell algorithm. Then, we searched for the hub genes in the co-expressed gene modules of high-abundance pDC and cDC patients with a weighted correlation network analysis (WGCNA). Finally, we revealed the impact of DCs on breast cancer and provide potential therapeutic strategies for precision breast cancer treatment. In short, our study elucidates the biological processes of DCs in breast cancer and provides new possibilities for identifying better therapeutic targets for breast cancer immunotherapy.

## 2. Results

### 2.1. High Abundance of pDCs and cDCs in Breast Cancer Is Associated with Better Survival Outcomes for pDCs More Significantly than for cDCs

**Figure 1:**
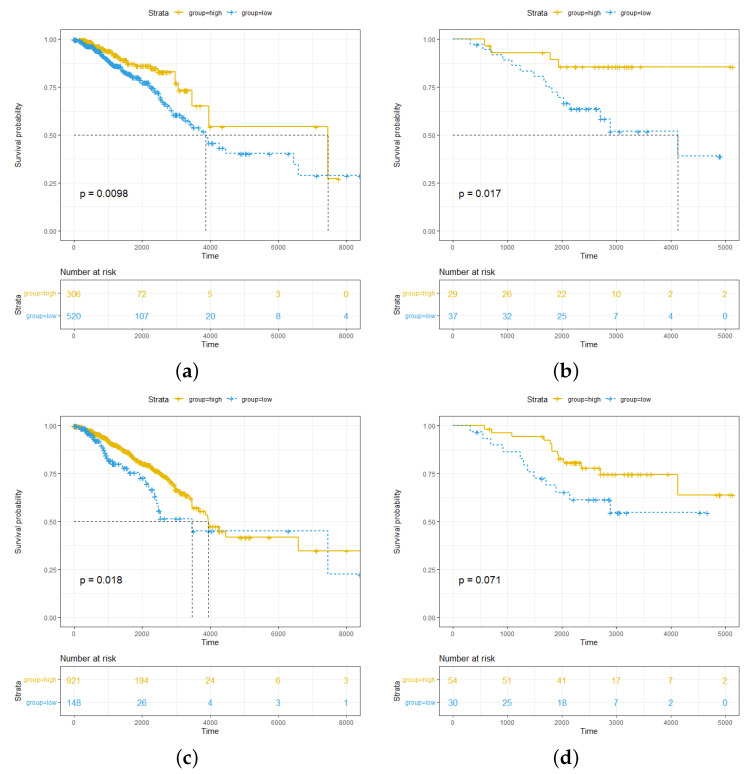
Correlation between clinical outcome and pDC or cDC abundance in breast cancer patients. (**a**) The Kaplan–Meier curves of breast cancer patients showed that patients with a high abundance of pDCs exhibited better prognoses in the TCGA cohorts. (**b**) The Kaplan–Meier curves of breast cancer patients showed that patients with a high abundance of pDCs exhibited better prognoses in the GSE20711 cohorts. (**c**) In the TCGA cohort, patients with a high abundance of cDCs also showed better prognoses. (**d**) In the GSE20711 cohort, the difference in the survival outcomes of the cDChigh and cDClow groups was not significant.

The cellular abundance of 64 immune and stromal cells was calculated for all samples in the TCGA dataset by using the xCell algorithm. For the pDC abundance results, samples with a result of 0 were removed, and the Kaplan–Meier survival curve was used to select the optimal threshold for dividing the remaining samples into the pDChigh and pDClow groups. Finally, the pDChigh group included 306 patients, and the pDClow group included 520 patients. The survival analysis results for the pDChigh and pDClow groups were significantly stratified (p=0.0098), and it was shown that a high abundance of pDCs was associated with better survival outcomes (Figure 1a). In addition, an external dataset (GSE20711) was used to validate the relationship between pDCs and breast cancer patient survival, and the results also had significant stratification (p=0.017) (Figure 1b).

The same method was used to divide the cDChigh and cDClow groups through thresholding in the TCGA dataset; finally, the cDChigh group included 921 patients, and the cDClow group included 148 patients. The survival analysis results in the cDChigh and cDClow groups were significantly stratified (p=0.018). This showed that a higher abundance of cDCs was associated with better survival outcomes (Figure 1c). However, this result was not significant in the external dataset (p=0.071) (Figure 1d).

### 2.2. Important Expression Modules for High-Abundance pDCs and cDCs

**Figure 2:**
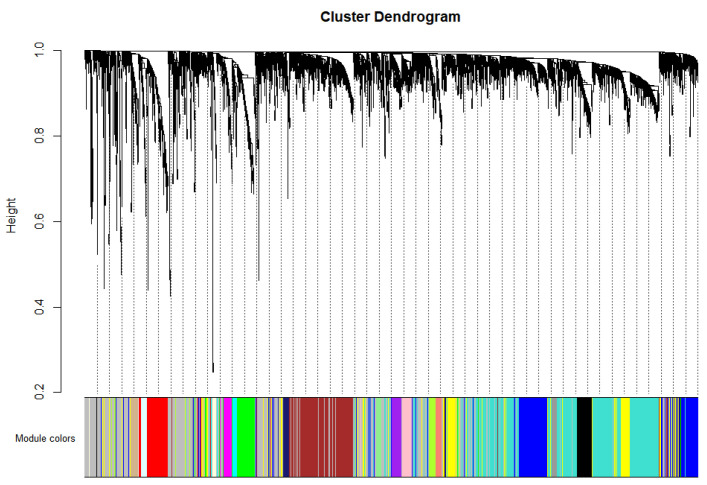
The hierarchical clustering tree of 21 co-expression modules.

We divided the tumor samples in the TCGA dataset into three categories: pDCs, cDCs, and others. Recent studies [14,15,16] and the results of Section 2.1 in this paper showed that pDC infiltration has a stronger correlation with survival in breast cancer patients than cDC infiltration. Therefore, in this study, all samples in the pDChigh group were categorized as “pDC”, the remaining cDChigh group samples that were not also included in the pDChigh group were categorized as “cDC”, and the samples that were in neither the pDChigh group nor the cDChigh group were categorized as “other”. After reading the fpkm expression matrix of the TCGA dataset and performing a $log_{2}\left(x+1\right)$ transformation, the top 5000 genes in MAD were screened out for follow-up studies. Finally, a one-step method was used to construct a weighted co-expression network, and a total of 21 co-expression modules were identified (Figure 2).

**Figure 3:**
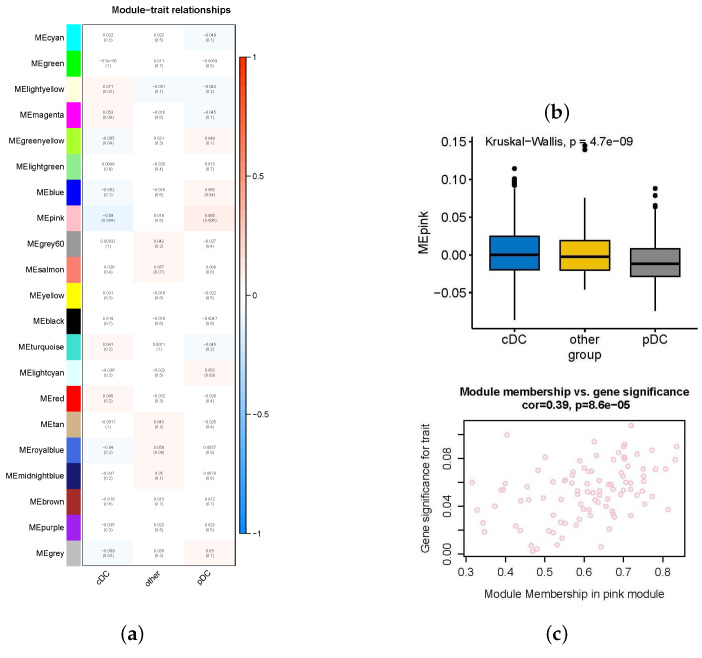
Correlations of gene modules with phenotypes. (**a**) Correlation heatmap of 21 gene modules with the phenotype, with the pink module being the most correlated with the phenotype. (**b**) The correlation boxplot of the pink module among the three phenotypes shows that there are significant differences in its expression. (**c**) The correlation scatterplot shows that the gene expression of the pink module and the correlation between the module and pDCs are significant.

The results showed that the pink module had the most significant correlation with the phenotype (Figure 3a). The expression of pink modules in these three phenotypes was significantly different (Figure 3b), and the correlation among gene expression in the module, the module, and pDCs was significant (Figure 3c). In the Figure 3, 4.7e−9 represents $4.7\times 10^{-9}$ and other data are similar in meaning. The same is true in the following section. Correlation boxplots for other modules and scatterplots of gene–module correlations for specific phenotypes are shown in Supplementary Figures S1 and S2.

### 2.3. Enrichment Analysis of Important Module Genes

**Figure 4:**
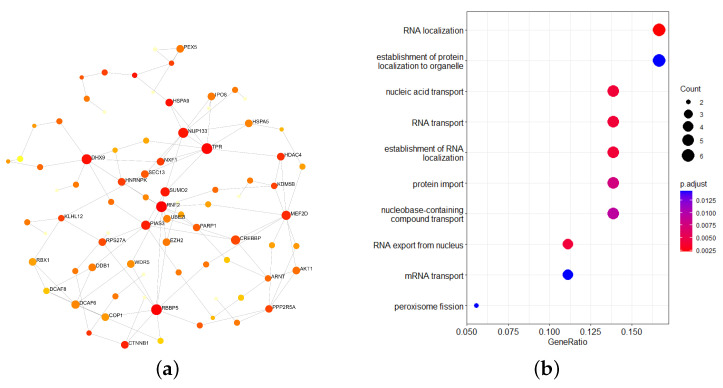
(**a**) The minimal network of the 97-gene PPI network of the pink module. (**b**) Visualization of the top ten items of the GO enrichment analysis results.

The 97 genes of the pink module were extracted to establish a protein–protein interaction (PPI) network. The minimum network of the PPI network contained 83 nodes in total, including 36 genes of the pink module (Figure 4a). A Kyoto Encyclopedia of Genes and Genomes (KEGG) enrichment analysis was performed on the screened genes by using the online website of the DAVID database, and all of the results of the enrichment analysis were selected with a significance of p<0.05. The results showed that these 36 genes were enriched in two pathways: hsa04146 and hsa03013 (refer Table 1).

After using the R package “org.Hs.eg.db” for a Gene Ontology (GO) enrichment analysis (Figure 4b), the results were mainly enriched in RNA localization, the establishment of protein localization in organelles, nucleic acid transport, RNA transport, the establishment of RNA localization, protein transport, and containing the compound transport of nucleobases.

### 2.4. RBBP5, HNRNPU, PEX19, TPR, and BCL9 Are Closely Related to Immunity

**Figure 5:**
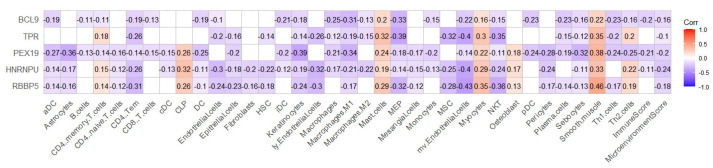
A heat map of the correlation between hub genes and immune cells. It can be seen that these five genes are closely related to the abundance of most immune cells.

Using the top 10 key genes of the PPI network of 36 pink module genes, two subnetworks were constructed with three network topology parameters: degree, betweenness, and closeness (Supplementary Figure S3). We screened five hub genes—*RBBP5*, *HNRNPU*, *PEX19*, *TPR*, and *BCL9*—and examined the correlation between the expression of these five hub genes and the level of immune cell infiltration (Figure 5). The expression of these five hub genes was closely related to the abundance of most immune cells in breast cancer patients, which also suggests that these five hub genes are closely related to immunity.

**Figure 6:**
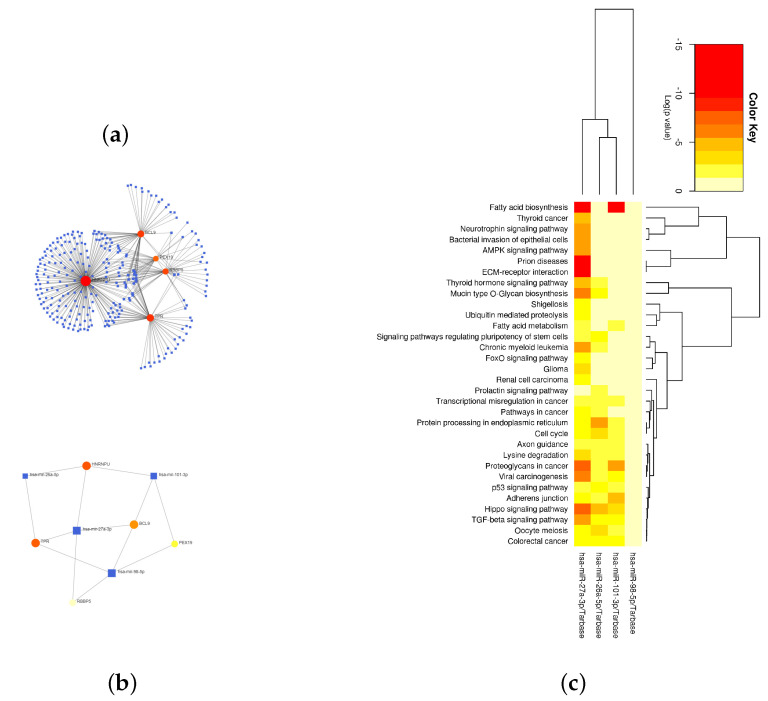
MiRNAs associated with the five hub genes. (**a**) The gene–miRNA interaction network of the five hub genes. (**b**) The minimal network of the gene–miRNA interaction networks of the five hub genes. (**c**) A heat map of the miRNA enrichment analysis results.

The gene–miRNA interaction network showed that these five hub genes were mainly closely related to the four miRNAs of hsa-miR-101-3p, hsa-miR-27a-3p, hsa-miR-26a-5p, and hsa-miR-98-5p (Figure 6a,b). The enrichment analysis of the target genes corresponding to the four miRNAs showed that the results were mainly enriched in fatty acid biosynthesis, prion diseases, the hippo signaling pathway, proteoglycans in cancer, etc. (Figure 6c).

### 2.5. Overexpression of RBBP5, TPR, and BCL9 Is Associated with Poor Clinical Breast Cancer Outcomes and the Wnt Pathway

**Figure 7:**
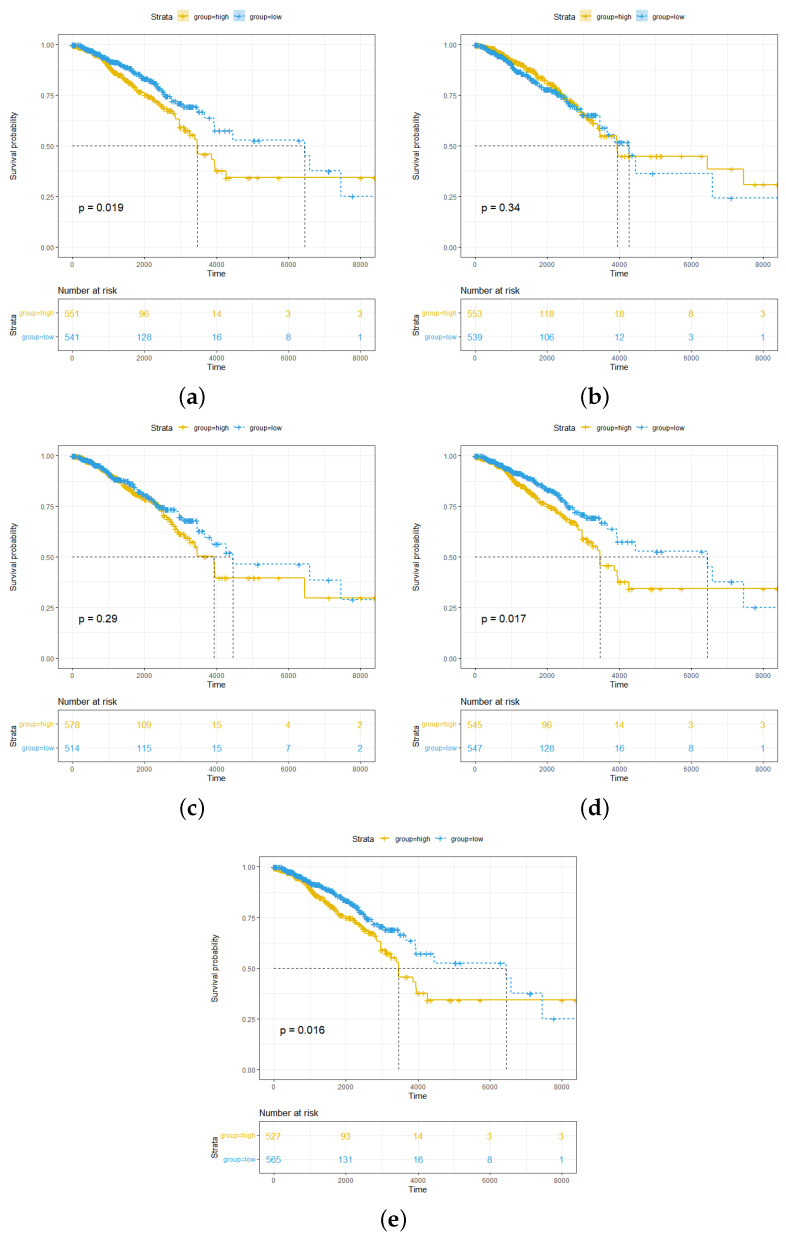
Correlation between clinical prognosis and hub gene expression in breast cancer patients. (**a**) Patients with low *RBBP5* expression have better prognoses. (**b**) *HNRNPU* expression is not significantly associated with patient prognosis. (**c**) *PEX19* expression is not significantly associated with patient prognosis. (**d**) Patients with low *TPR* expression have better prognoses. (**e**) Patients with low *BCL9* expression have better prognoses.

We further analyzed the effect between the different expression levels of the five hub genes and survival (Figure 7a–e). The results showed that in breast cancer patients, overexpression of *RBBP5*, *TPR*, and *BCL9* was significantly associated with poor clinical outcomes (p<0.05). Two other hub genes (*HNRNPU* and *PEX19*) did not show significant prognostic value.

**Figure 8:**
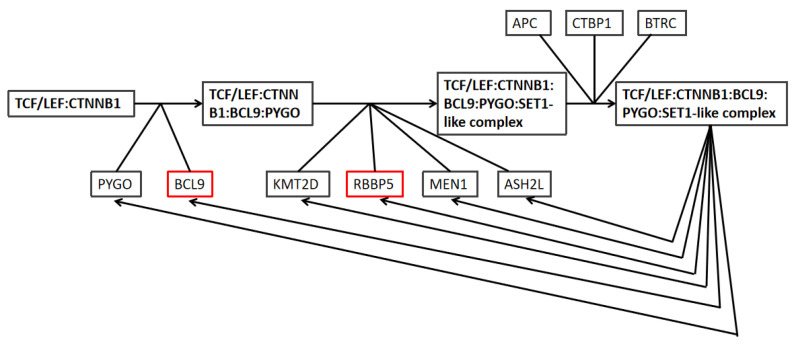
Gene pathways in response to TCF-related instructions of the Wnt pathway. The red boxes indicate the locations of *RBBP5* and *BCL9*.

The Reactome database was used to search for the related pathways of three genes—*RBBP5*, *TPR*, and *BCL9*—and the results showed that *RBBP5* and *BCL9* are jointly involved in responding to the TCF-related instructions of the Wnt pathway (Figure 8). The general Wnt pathway mainly refers to the classical signaling pathway mediated by β-catenin, which further promotes the maintenance of cancer by mediating immune escape and immunotherapy resistance. The signaling pathways of *RBBP5* and *BCL9* showed their involvement in both the formation of β-catenin and the deactivation of the β-catenin transactivating complex. The Wnt signaling pathway is a complex network of protein interactions that functions most commonly in embryonic development and cancer, but is also involved in normal physiological processes in adult animals [17]. The signaling pathways revealed that *BCL9* associates with TCF, and *RBBP5* recruits the SET1 methyltransferase complex to promote β-catenin formation. Meanwhile, both *RBBP5* and *BCL9* are involved with adenomatous polyposis Escherichia coli (APC) in the promotion of the phosphorylation and degradation of β-catenin, thereby inhibiting the Wnt signaling pathway.

### 2.6. Patients with High Abundance of pDCs and cDCs Are More Sensitive to Chemotherapy Drugs

**Figure 9:**
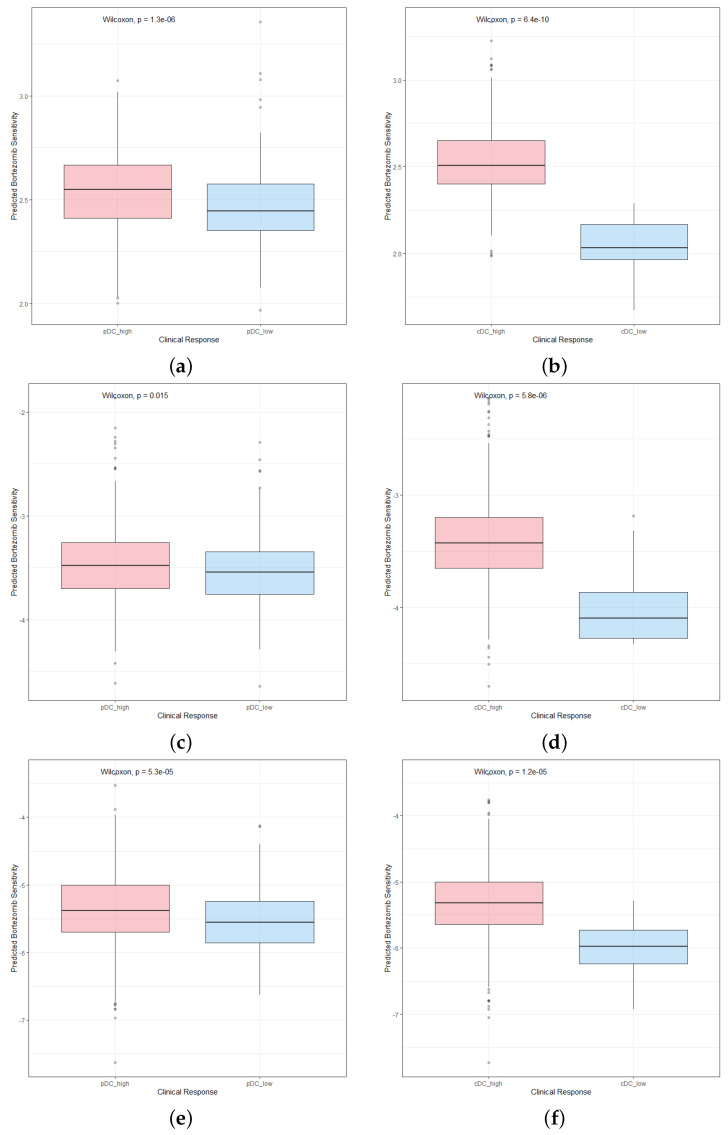
Visualization of the differences in the sensitivities of breast cancer patients with different DC abundances to chemotherapeutics. (**a**,**b**) Breast cancer patients with a high abundance of pDCs and cDCs were more sensitive to lapatinib. (**c**,**d**) Breast cancer patients with a high abundance of pDCs and cDCs were more sensitive to paclitaxel. (**e**,**f**) Breast cancer patients with a high abundance of pDCs and cDCs were more sensitive to docetaxel.

Chemotherapy is a common treatment for breast cancer. We used three chemotherapeutic agents—lapatinib, paclitaxel, and docetaxel—to assess the responses of pDCs and cDCs of varying abundances to chemotherapy. First, we downloaded the cell line data from the GDSC database. Then, we trained the prediction model by using ridge regression and determined the prediction accuracy through 10-fold cross-validation. Finally, the prediction model was used to calculate each patient’s response to drug sensitivity to lapatinib, paclitaxel, and docetaxel in each group. The results showed that the drug sensitivities of high- and low-abundance pDCs and cDCs to lapatinib, paclitaxel, and docetaxel were significantly different, and the high-abundance group was more sensitive (Figure 9a–e). We calculated the fold changes in the drug sensitivities of patients in different groups (refer Table 2). This also showed that a high abundance of pDCs and cDCs had a better effect on the prognosis and treatment of breast cancer, and a difference in DC abundance had a greater influence on the therapeutic effect of lapatinib than on that of paclitaxel and docetaxel. In addition, the abundance of cDCs had a greater effect on the chemotherapeutic efficacy of lapatinib than that of pDCs did, but it had less of an effect on paclitaxel and docetaxel.

After calculating the IC50 values of the pDChigh group and the cDChigh group, they were sorted from small to large according to the IC50 values. The results showed that bortezmib, dactinomycin, and docetaxel were the most effective drugs (refer to Table 3, Supplementary Tables S1 and S2). Among them, bortezomib is used for the treatment of patients with multiple myeloma. Dactinomycin acts on mRNA to interfere with the transcription process of cells, and when used in combination with radiation, it can increase the sensitivity of tumors to radiation. Dactinomycin can clinically treat diseases such as Wilms tumor, rhabdomyosarcoma, and neuroblastoma. The effect of docetaxel is the same as that of paclitaxel. It is a specific drug for the mitotic period and has a good effect on advanced breast cancer, ovarian cancer, and non-small-cell lung cancer.

## 3. Discussion

DCs, as the “command officers” of the immune system, play an important role in the process of antitumor immunity. DC-targeted vaccines have been developed in many clinical trials to improve tumor immunotherapy [18]. However, as they are new members of the DC family, there are many controversies about the specific role of pDCs, and there are few articles that have studied pDCs and cDCs together.

We searched for new biomarkers from a co-expression module of pDCs and cDCs, and we screened five hub genes from the module: *RBBP5*, *HNRNPU*, *PEX19*, *TPR*, and *BCL9*. *Retinoblastoma-binding protein 5 (RBBP5)* is one of the core subunits regulating mixed-lineage leukemia protein 1 (MLL1) methyltransferase activity [19]. Studies have shown that an increase in *RBBP5* in cancer stem cells can inhibit the growth of glioblastoma [20]. The *TPR* (tetratripeptide repeat) is a class of genes containing TPR-conserved motifs [21]. This motifs can inhibit viral particle release and regulate mitochondrial fission [22]. Yang et al. found that the *TPR* promotes the proliferation of hepatocellular carcinoma cells by promoting cytokinesis [23]. Du et al. revealed that the *TPR* is overexpressed in breast cancer cells [24]. *B-cell lymphoma 9 (BCL9)* is a key transcriptional cofactor of β-catenin [25]. Behbod et al. found that *BCL9* is a molecular driver of the aggressive progression of ductal carcinoma in situ and may predispose one to basal invasive breast cancer [26]. These studies showed that all of the pivotal genes found in this paper play an important role in tumor therapy. Among them, *BCL9* was proven to be closely related to DCs in breast cancer [27], while the *TPR* and *RBBP5* were shown to be closely related to DCs, but there is a lack of further research on breast cancer [28,29]. For the first time, this paper proposes that *HNRNPU* and *PEX19* are related to the prognosis of DCs in cancer, which also provides new possibilities for finding new targets for breast cancer immunotherapy.

Our results also show that *RBBP5* and *BCL9* are involved in responding to the TCF-related instructions of the Wnt pathway. Recent studies showed that the deletion of *BCL9* and *BCL9l* in colorectal cancer was found to inhibit colon tumorigenesis driven by an abnormal Wnt signaling pathway [30]. However, our results show that the low expression of *RBBP5* and *BCL9* is related to a better prognosis of breast cancer, which indicates that the abnormal Wnt pathway may also be inhibited by reducing the expression of *RBBP5* and *BCL9* in breast cancer, thus achieving better targeted therapy.

In addition, chemotherapy is a commonly used method in the clinical treatment of breast cancer. Therefore, we predicted the chemotherapy response of the divided high- and low-abundance groups to further verify the importance of dendritic cells in the prognosis and treatment of breast cancer patients. However, the therapeutic effects of these drugs on breast cancer and their mechanisms of action in combination with other drugs require further research.

Current studies have shown that the abundance of DCs is closely related to the prognosis of cancer, but the prognostic effect is different for different cancers [31,32]. Therefore, it was more targeted for us to analyze breast cancer patients separately in this paper. Of course, the method in this paper can also be used to study other cancers, and we believe that there will be more new conclusions.

## 4. Materials and Methods

### 4.1. Database

We downloaded a total of 1217 breast cancer patients’ gene expression profiles from The Cancer Genome Atlas (TCGA) based on the cancer genomics data analysis platform UCSC Xena (https://xena.ucsc.edu/, accessed on 17 June 2022) [33]. By integrating clinical data such as age, grade, survival status, and survival time, a total of 1069 cases of breast cancer patients were obtained. Then, the gene expression profiles (GSE20711) [34] and clinical information of 90 breast cancer patients were retrieved from the Gene Expression Omnibus (GEO) (https://www.ncbi.nlm.nih.gov/geo/ accessed on 3 July 2022). For the above datasets, we converted the Ensemble ID into a gene symbol and averaged the values when multiple probes were mapped to the same gene.

### 4.2. Calculation of Immune-Activity-Related Cellular Abundance

The cellular abundance of 64 immune and stromal cells was calculated for all tumor samples in the TCGA and GEO datasets by using the xCell algorithm [35]. The principle of the algorithm is to extract the signatures of 64 immune cells and stromal cells by using a machine learning algorithm and convert the enrichment scores into cell-type scores. Compensation corrections were finally made for closely related cell-type fractions.

### 4.3. Survival Analysis of pDC and cDC Infiltration

After calculating the cell infiltration with the xCell algorithm, we merged the infiltration-level data and survival data of the patients from the TCGA and GEO datasets. The Kaplan–Meier survival curve was used to determine the high- and low-abundance thresholds of pDCs and cDCs, and the TCGA dataset was divided into the pDChigh and pDClow groups, as well as the cDChigh and cDClow groups. At the same time, the GEO dataset was used as a validation set to verify the prognostic performance of pDCs and cDCs.

### 4.4. Searching for Co-Expressed Gene Modules with WGCNA

WGCNA was used to find co-expressed gene modules in the pDChigh group and cDChigh group. We identified a total of 21 co-expression modules, which were marked with different colors by setting a soft threshold power of 4, which represented genes that shared highly similar expression patterns in breast cancer patients with high-abundance pDCs and cDCs. Important modules were selected by plotting heat maps of module–phenotype correlations, correlation box plots, and scatterplots of gene–module correlations for specific phenotypes. Finally, the genes in the module were exported.

### 4.5. Enrichment Analysis of Important Modules

A PPI network of important modules was constructed with the STRING database and NetworkAnalyst3.0 with a confidence score of 900. Finally, a minimal network was established. GO and KEGG analyses were used for gene set annotation.

### 4.6. Looking for Hub Genes of Important Modules and Their Associated miRNAs

First, based on the minimum PPI network of genes in important modules, three network topology parameters—degree, betweenness, and closeness—were used to screen key genes; then, the cytoHubba plug-in was used to construct the relationship network of the top 10 key genes, and the hub genes were screened out. Then, for the selected hub gene, the correlation between its expression and the level of immune cell infiltration was first calculated to show whether the hub gene was related to immunity. Finally, a gene–miRNA interaction network of hub genes was constructed with the Tarbase database and NetworkAnalyst3.0, and the main miRNAs were enriched by miRpath (http://www.microrna.gr/miRPathv3/ accessed on 22 September 2022).

### 4.7. Survival Analysis and Pathway Analysis of Hub Genes

Survival analysis was performed on the selected hub genes to show whether they had prognostic value. The related pathways of hub genes with significant prognostic value were searched in the Reactome database (https://reactome.org/ accessed on 20 September 2022) [36] to find their mechanisms of action.

### 4.8. Chemotherapy Response Prediction and Drug Screening

Based on the largest public pharmacogenetics database, the Genomics of Drug Sensitivity in Cancer (GDSC) database, we used the R package “pRRophetic” to calculate the sensitivity of samples with different abundances of pDCs and cDCs to chemotherapy drugs in the TCGA dataset [37]. In this way, the relationship between the abundance of pDCs and cDCs and the response to chemotherapy could be predicted. Then, we used the R package “oncoPredict” to perform drug prediction, calculated the drug IC50 value of each sample in the pDChigh group and the cDChigh group, and took the average of the IC50 values of all samples as the final drug IC50 value.

### 4.9. Statistical Analysis

Statistical analysis was carried out with the R software (version 4.1.2). Immune-activity-related cell abundance was calculated by using the xCell package. Kaplan–Meier survival curve mapping was carried out with the survival and survminer packages. The WGCNA package was used for the WGCNA analysis, and the ggpubr package was used for plotting. Enrichment analysis was performed by using the clusterProfiler and org.Hs.eg.db packages. The correlation between the hub gene expression and immune cell infiltration levels was calculated by using the psych package. For all of the results, p<0.05 is considered to be statistically significant.

## 5. Conclusions

This research provided a potential therapeutic strategy for the precise treatment of breast cancer by dividing patients into four groups according to the infiltration of pDCs and cDCs and searching the co-expressed gene modules in high-abundance pDC and cDC patients through WGCNA. We finally found five hub genes, among which three hub genes are significantly related to the prognosis of breast cancer patients. The results also showed that some biomarkers are involved in the response of TCF-related instructions in the Wnt pathway. Their role in the Wnt pathway and their relationship with dendritic cells deserve further discussion, which will be a very worthwhile and meaningful direction to explore. In addition, our research on the discovered drugs is only the beginning, and their therapeutic effects on breast cancer and the mechanisms of their combination with other drugs need further study.

## Figures and Tables

**Table 1:** KEGG enrichment analysis results for 36 genes in the minimal network.

Category	Term	Genes	*p* Value
KEGG	hsa04146:Peroxisome	*GNPAT*, *PEX19*, *PEX11B*	1.6 × 10^−2^
KEGG	hsa03013:Nucleocytoplasmic transport	*IPO9*, *NUP133*, *TPR*	2.8 × 10^−4^

**Table 2:** Calculation of the fold changes in the sensitivities of the pDC and cDC groups to three chemotherapeutic drugs.

Pharmaceuticals	pDC_FoldChange	cDC_FoldChange
Lapatinib	1.021542892	1.157434474
Paclitaxel	0.982754003	0.893367891
Docetaxel	0.976301981	0.917481043

**Table 3:** Top five drugs with the lowest IC50 values for drug prediction outcomes in patients with a high abundance of pDCs and cDCs.

Pharmaceuticals	pDChigh_Average	cDChigh_Average
Bortezmib_1191	0.0087	0.0086
Dactinomycin_1911	0.0103	0.0102
Docetaxel_1007	0.0145	0.0141
Daporinad_1248	0.0153	0.0152
Sepantronium.bromide_1941	0.0154	0.0155

## Data Availability

This work is based on a secondary analysis of publicly available datasets. Informed consent was not required. The data used and analyzed during the current study are available from UCSC Xena and the Gene Expression Omnibus (GEO). The datasets generated and analyzed in the current study are available from the corresponding author upon reasonable request.

## Supplementary Materials

The following supporting information can be downloaded at: https://www.mdpi.com/article/10.3390/ijms24044058/s1.

## Abbreviations

The following abbreviations are used in this manuscript:

DC	Dendritic Cell
cDC	Conventional Dendritic Cell
pDC	Plasmacytoid Dendritic Cell
WGCNA	Weighted Correlation Network Analysis
PPI	Protein–Protein Interaction
GO	Gene Ontology
KEGG	Kyoto Encyclopedia of Genes and Genomes
