# Supplementary material for: New Biomarkers Based on Dendritic Cells for Breast Cancer Treatment and Prognosis Diagnosis
Source: Int J Mol Sci. 2023 Feb 17;24(4):4058. doi: 10.3390/ijms24044058 (PMC9963148; doi:10.3390/ijms24044058)
Supplement: Supplementary file 1 [file ijms-24-04058-s001.zip › Supplementary Figures and Tables legends.pdf]

Figures S1 and S2 are the relevant box charts of all modules and the relevant scatter charts of gene modules with specific phenotypes.
Supplementary Figures S3 are two hub gene sub-networks based on PPI network of pink module gene.
Supplementary Tables S1 and S2 are all IC50 values of pDChigh group and cDChigh group respectively.
