# Supplementary figures and images for: New Biomarkers Based on Dendritic Cells for Breast Cancer Treatment and Prognosis Diagnosis
Source: Int J Mol Sci. 2023 Feb 17;24(4):4058. doi: 10.3390/ijms24044058 (PMC9963148; doi:10.3390/ijms24044058)

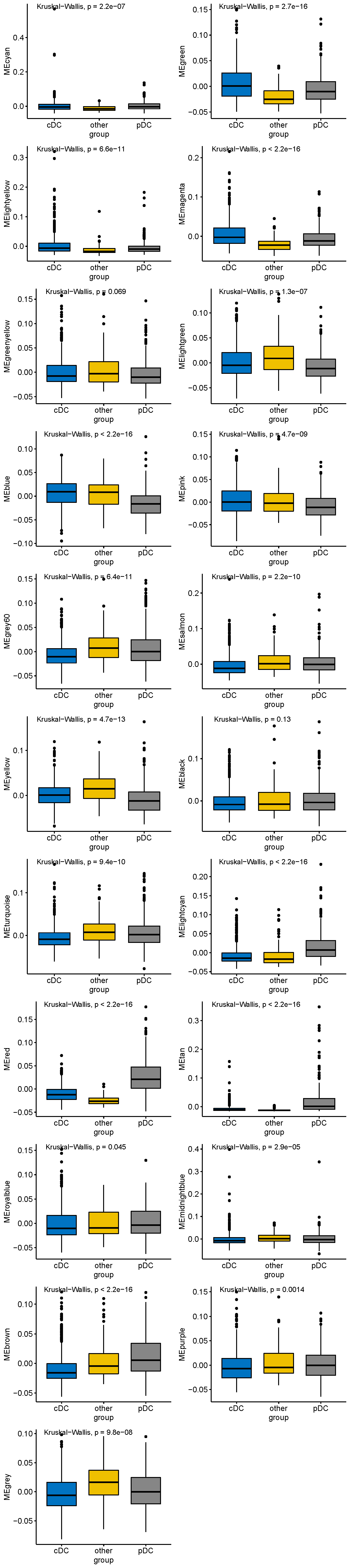

Supplement: Supplementary file 1 [file ijms-24-04058-s001.zip › Supplementary Figure S1.png]

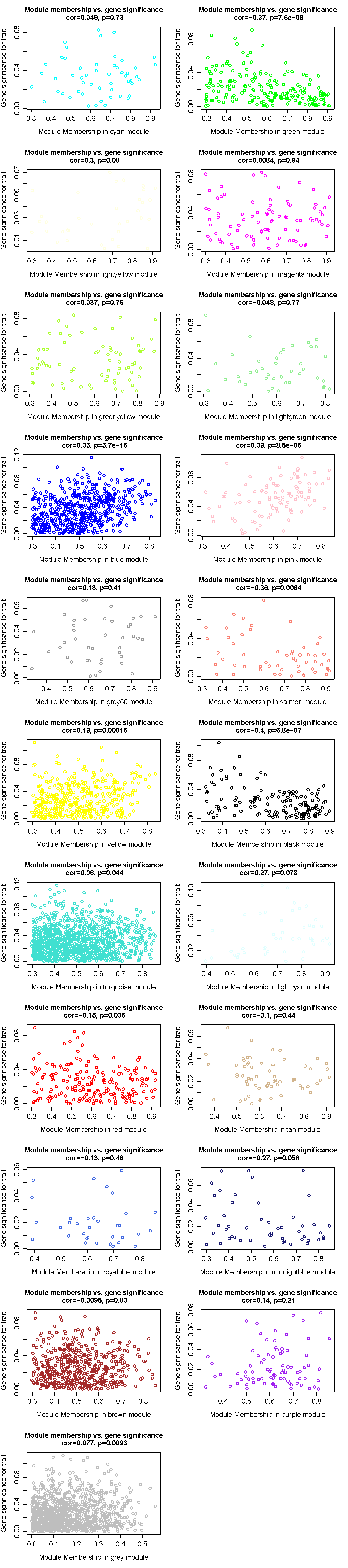

Supplement: Supplementary file 1 [file ijms-24-04058-s001.zip › Supplementary Figure S2.png]

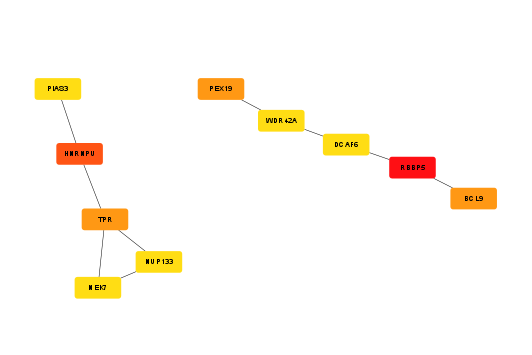

Supplement: Supplementary file 1 [file ijms-24-04058-s001.zip › Supplementary Figure S3.png]
